# Supplementary material for: Photochromism from wavelength-selective colloidal phase segregation
Source: Nature. 2023 May 17;617(7961):499–506. doi: 10.1038/s41586-023-05873-4 (PMC10191859; doi:10.1038/s41586-023-05873-4)
Supplement: Supplementary file 1 — This file contains Supplementary Figs. 1–6 and Tables 1–5. [file 41586_2023_5873_MOESM1_ESM.pdf]

nature portfolio

https://doi.org/10.1038/s41586-023-05873-4

Supplementary information

Photochromism from wavelength-selective colloidal phase segregation

In the format provided by the authors and unedited

1. **Supplementary Figures**

Fig. S1 Three-dimensional phase segregation of ternary colloidal system....................................P2

Fig. S2 Spectra-selective active colloidal mixture as photochromic colloidal swarm..................P3

Fig. S3 The scanning electron microscopy (SEM) image of monodispersed TiO2 microbeads...P3

Fig. S4 Molecular structure and absorption spectrum of organic dyes...P4

Fig. S5 Radial distribution function of dye-sensitized-colloid modulated with illumination......P4

Fig. S6 Apparent pair potential of dye-sensitized-colloid modulated with illumination............P5

2. **Supplementary Tables**

Table S1 Fitting parameters under various blue light illumination..P6

Table S2 Fitting parameters under various red light illumination...P6

Table S3 Expected layered phase behaviour under different illumination spectra................................P6

Table S4 The zeta potential of pristine TiO_2 and different dye-loaded TiO_2..P7

Table S5 Comparison of current state-of-the-art photochromic materials..P7

1. Supplementary Figures

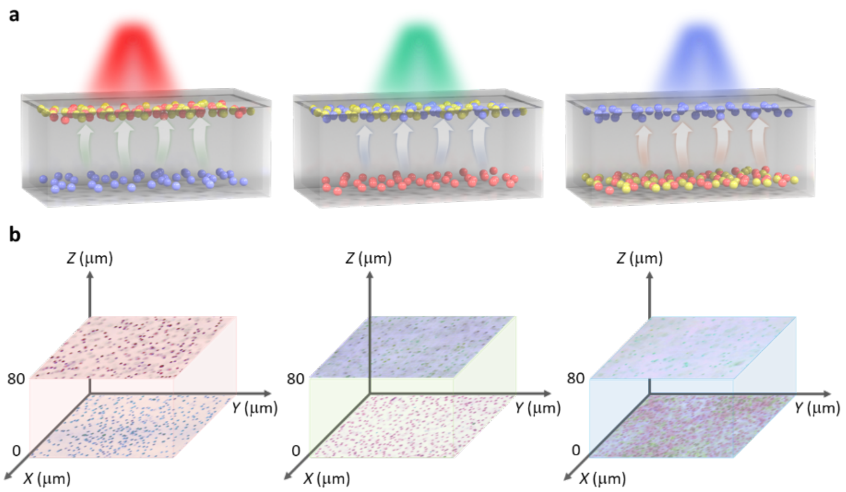

Fig. S1| Three-dimensional phase segregation of ternary colloidal system a, The schematic of three-dimensional phase segregation of the ternary colloidal system. A mixture solution of L0, LEG4, and SQ2 sensitized TiO_2 colloids is transferred into a transparent chamber. Different light illumination induces various vertical segregation. **b,** 3D distribution of the coloured colloids at different depths (Z=0, 80 μm) under red, green, and blue light illumination, respectively. L0 and LEG4 sensitized TiO_2, SQ2, and L0 sensitized TiO_2, SQ2 sensitized TiO_2 are pushed up to the top under red, green, and blue light illumination, respectively.

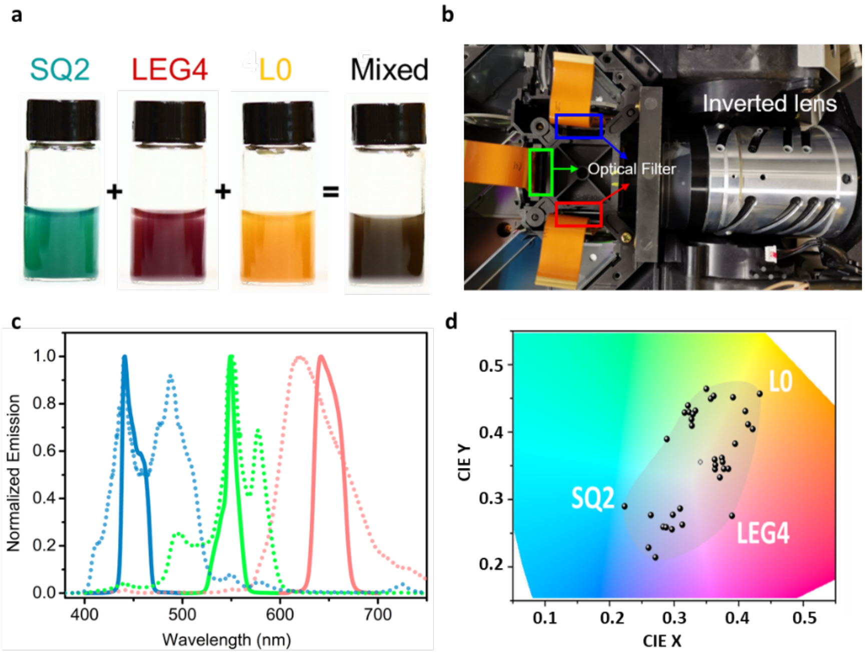

Fig. S2| Spectra-selective active colloidal mixture as photochromic colloidal swarm. a, The photograph of SQ2, LEG4, and L0 sensitized TiO_2 colloids and their mixed suspension, which appear as cyan, magenta, yellow and black ink, respectively. **b,** Modified 3LCD projector with optical filters. **c,** The normalized spectrum of the projector output for blue, green, and red light with (solid line) or without (dash line) optical filter. **d,** Experimental colour gamut of the photochromic colloidal swarm presented in a standard CIE-1931 colour space.

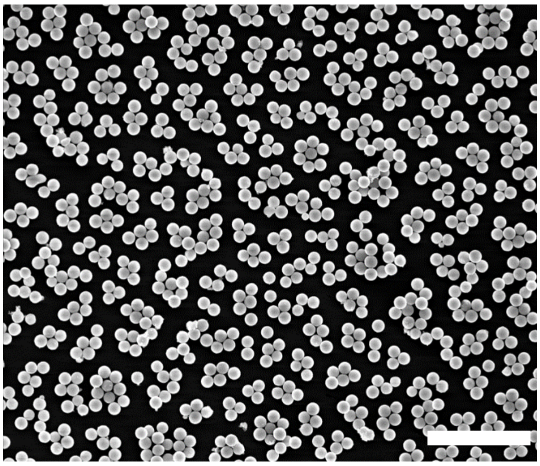

Fig. S3| The scanning electron microscopy (SEM) image of monodispersed TiO_2 microbeads. Scale bars: 10 μm.

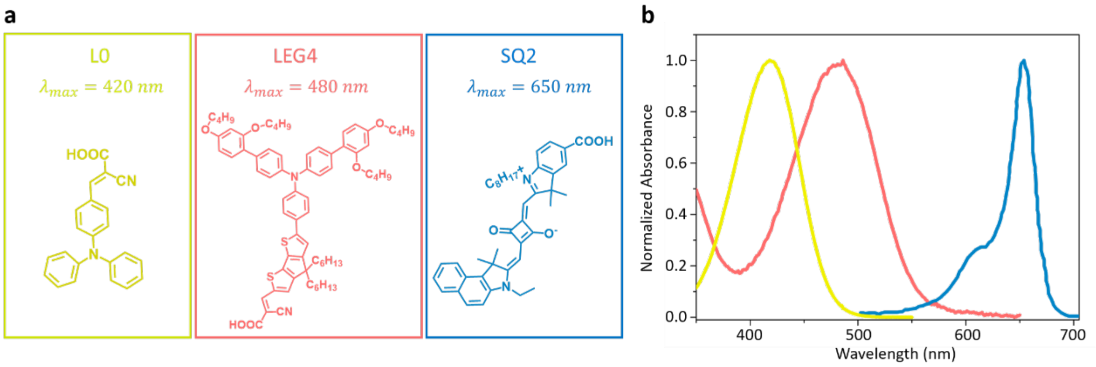

Fig. S4| Molecular structure and absorption spectrum of organic dyes a, The molecular structure and maximum absorption wavelength of dye L0, LEG4, and SQ2. **b,** The normalized absorbance of ethanolic solution of dye (L0 (yellow), LEG4 (magenta), and SQ2 (cyan)), with distinctive absorbance covering the visible spectrum.

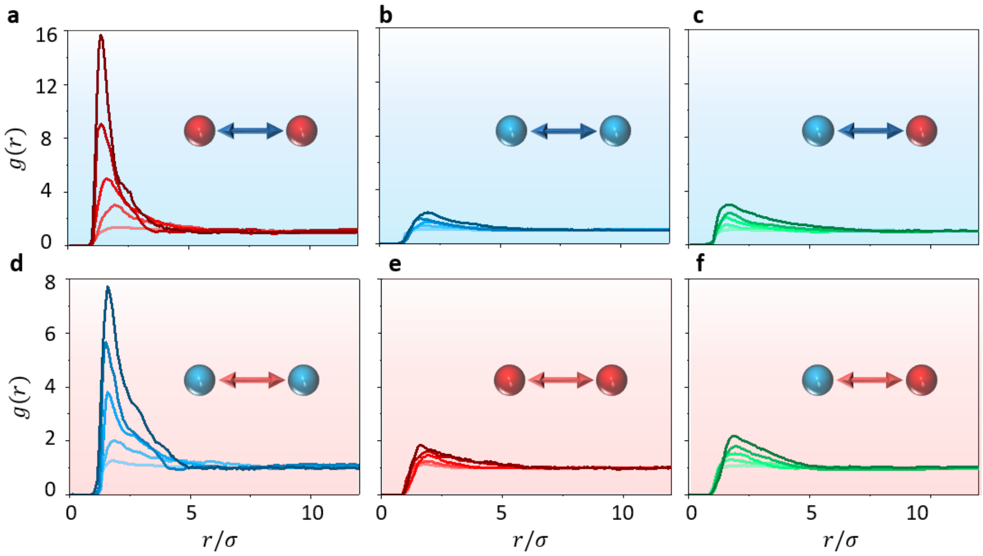

Fig. S5| Radial distribution function of dye-sensitized-colloid modulated with illumination. a-c, The $g(r)$ as a function of r/σ for LEG4-LEG4 sensitized TiO_2 colloids (**a**), SQ2-SQ2 sensitized TiO_2 colloids (**b**), and LEG4-SQ2 sensitized TiO_2 colloids (**c**) under blue light (440-480 nm) illumination with various intensity (10 mW/cm^2, 30 mW/cm^2, 50 mW/cm^2, 70 mW/cm^2, 90 mW/cm^2). **d-f,** The $g(r)$ for SQ2-SQ2 sensitized TiO_2 colloids (**d**), LEG4-LEG4 sensitized TiO_2 colloids (**e**), and LEG4-SQ2 sensitized TiO_2 colloids (**f**) under red light (640-660 nm) illumination with various intensity (10 mW/cm^2, 30 mW/cm^2, 50 mW/cm^2, 70 mW/cm^2, 90 mW/cm^2).

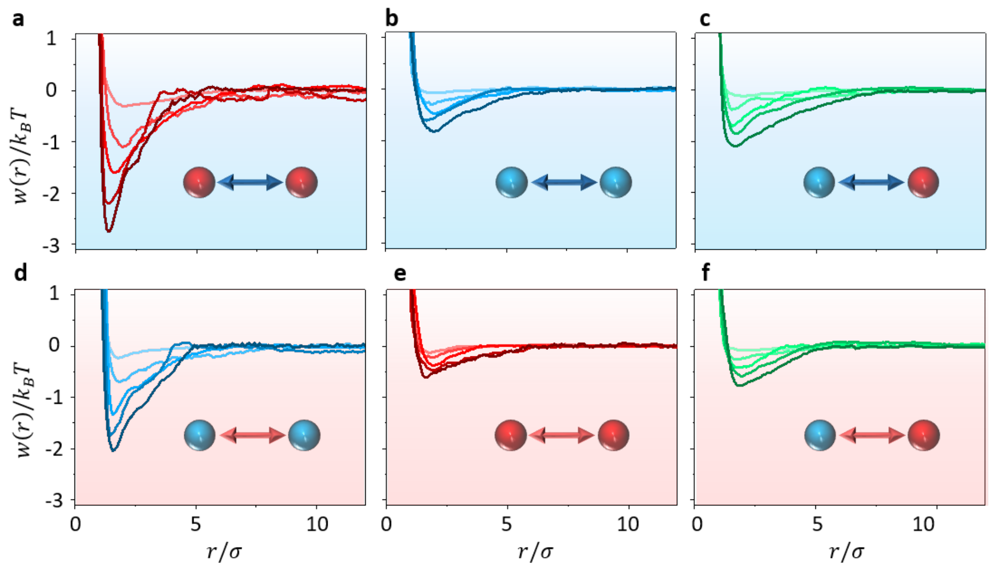

Fig. S6| Apparent pair potential of dye-sensitized-colloid modulated with illumination. a-c, The $w(r)$ as a function of r/σ for LEG4-LEG4 sensitized TiO_2 colloids (**a**), SQ2-SQ2 sensitized TiO_2 colloids (**b**), and LEG4-SQ2 sensitized TiO_2 colloids (**c**) under blue light (440-480 nm) illumination with various intensity (10 mW/cm^2, 30 mW/cm^2, 50 mW/cm^2, 70 mW/cm^2, 90 mW/cm^2). **d-f,** The $w(r)$ for SQ2-SQ2 sensitized TiO_2 colloids (**d**), LEG4-LEG4 sensitized TiO_2 colloids (**e**), and LEG4-SQ2 sensitized TiO_2 colloids (**f**) under red light (640-660 nm) illumination with various intensity (10 mW/cm^2, 30 mW/cm^2, 50 mW/cm^2, 70 mW/cm^2, 90 mW/cm^2).

2. Supplementary Tables:

Table S1. Fitting parameters under various blue light illumination

passive-passive	10 mW/cm^2	30 mW/cm^2	50 mW/cm^2	70 mW/cm^2	90 mW/cm^2
D_0 (k_BT)	0.1086	0.3643	0.5357	0.6894	0.8786
α (µm^{-1})	2.6526	1.7027	1.2636	1.3329	0.9301
r (µm)	1.5975	1.6775	1.9440	1.8631	2.0373
**active-active**	**10 mW/cm^2**	**30 mW/cm^2**	**50 mW/cm^2**	**70 mW/cm^2**	**90 mW/cm^2**
D_0 (k_BT)	0.4000	1.2503	1.7840	2.2361	2.7269
α (µm^{-1})	1.6438	1.1023	1.1132	1.4569	1.3855
r (µm)	1.7563	1.9024	1.7306	1.5977	1.5193
**passive-active**	**10 mW/cm^2**	**30 mW/cm^2**	**50 mW/cm^2**	**70 mW/cm^2**	**90 mW/cm^2**
D_0 (k_BT)	0.2084	0.4070	0.6449	0.8762	1.2472
α (µm^{-1})	2.1335	1.1827	1.1901	1.0952	0.9000
r (µm)	1.6116	1.9551	1.7884	1.8615	1.9441

Table S2. Fitting parameters under various red light illumination

passive-passive	10 mW/cm^2	30 mW/cm^2	50 mW/cm^2	70 mW/cm^2	90 mW/cm^2
D_0 (k_BT)	0.1212	0.2268	0.3492	0.5339	0.6908
α (µm^{-1})	2.6771	1.6444	1.3447	1.1297	1.2802
r (µm)	1.6707	1.7449	1.9612	1.8925	1.8015
**active-active**	**10 mW/cm^2**	**30 mW/cm^2**	**50 mW/cm^2**	**70 mW/cm^2**	**90 mW/cm^2**
D_0 (k_BT)	0.3016	0.7787	1.2406	1.6579	2.0594
α (µm^{-1})	1.2958	1.1339	1.1692	1.4006	1.1553
r (µm)	2.2428	2.0770	1.9855	1.8078	1.7847
**passive-active**	**10 mW/cm^2**	**30 mW/cm^2**	**50 mW/cm^2**	**70 mW/cm^2**	**90 mW/cm^2**
D_0 (k_BT)	0.1184	0.3530	0.4935	0.6431	0.8430
α (µm^{-1})	2.3619	1.7563	1.5617	1.2230	1.0367
r (µm)	1.6193	1.7640	1.7081	1.8746	2.0277

Table S3. Expected layered phase behaviour under different illumination spectra

Illuminated light	Light component	Top layer (passive)	Bottom layer (active)
Red	Red	L0-TiO_2 + LEG4-TiO_2	SQ2-TiO_2
Green	Green	L0-TiO_2 + SQ2-TiO_2	LEG4-TiO_2
Blue	Blue	SQ2-TiO_2	L0-TiO_2 + LEG4-TiO_2
Cyan	Green + Blue	SQ2-TiO_2	L0-TiO_2 + LEG4-TiO_2
Magenta	Red + Blue	LEG4-TiO_2 (weak active)	L0-TiO_2 + SQ2-TiO_2
Yellow	Red + Green	L0-TiO_2	LEG4-TiO_2 + SQ2-TiO_2

Table S4. The zeta potential of pristine TiO_2 and different dye-loaded TiO_2

Zeta Potential (mV)				
	pristine	L0-TiO_2	LEG4-TiO_2	SQ2-TiO_2
(+)TiO_2	+10.33	+8.23	+7.78	+8.72
(-)TiO_2	-38.45	-42.38	-48.68	-15.01

Table S5. Comparison of current state-of-the-art photochromic materials.

Type of Materials	Excitation Light	Response Time	Display Color	Mechanism	Ref.
Photochromic Colloidal Swarm	Full Spectrum (~10-20 mW/cm^2)	1 min	Full Spectrum (positive and negative response) No Erase needed	Phase Segregation	This Paper
Phosphomolybdic Acid	365 nm UV Flashlight (5 W)	5 min	Dark Blue Air oxidation erase	Valence Transition	[55]
Poly(ionic liquids) and Polyoxometalates	Projector Lamp (210 W)	2 min	Light Yellow and Bluish Green Air oxidation erase	Valence Transition	[56]
Bismuth Oxyhalide	365 nm UV (6 mW/cm^2)	1 min	Brown Black Air oxidation erase	Valence Transition	[57]
WO_3 Nanoparticles	UV lamp (5 W)	3 min	Blue Heat erase	Valence Transition	[58]
Photochromic Diarylethene Derivatives	254 nm UV (1 mW/cm^2)	30 min	Cyan or Magenta or Yellow Visible light erase	Isomerization	[59]
Photochromic Dyes	Full Spectrum (1200 Lumens)	20-120 min	Full Spectrum (positive response) UV (4W) erase	Isomerization	[60]
